# Effects of different stimulators of cGMP synthesis on lipid content in bovine oocytes matured *in vitro*

**DOI:** 10.1590/1984-3143-AR2021-0072

**Published:** 2021-12-10

**Authors:** Letícia Schefer, Kátia Regina Lancelloti Schwarz, Daniela Martins Paschoal, Fernanda Cavallari de Castro, Hugo Fernandes, Ramon César Botigelli, Cláudia Lima Verde Leal

**Affiliations:** 1 Departamento de Medicina Veterinária, Faculdade de Zootecnia e Engenharia de Alimentos, Universidade de São Paulo, Pirassununga, SP, Brasil

**Keywords:** natriuretic peptides, NPR1, guanylate cyclase, lipid droplets, Nile red

## Abstract

Bovine oocytes and blastocysts produced *in vitro* are frequently of lower quality and less cryotolerant than those produced *in vivo*, and greater accumulation of lipids in the cytoplasm has been pointed out as one of the reasons. In human adipocytes cGMP signaling through the activation of PKG appears to be involved in lipid metabolism, and components of this pathway have been detected in bovine cumulus-oocyte complexes (COCs). The aim of this study was to investigate the influence of this pathway on the lipid content in oocytes and expression of PLIN2 (a lipid metabolism-related gene) in cumulus cells. COCs were matured *in vitro* for 24 h with different stimulators of cGMP synthesis. The activation of soluble guanylyl cyclase (sGC) by Protoporphyrin IX reduced lipid content (22.7 FI) compared to control oocytes (36.45 FI; P <0.05). Stimulation of membrane guanylyl cyclase (mGC) with natriuretic peptides precursors A and C (NPPA and NPPC) had no effect (36.5 FI; P>0.05). When the PKG inhibitor KT5823 was associated with Protoporphyrin IX, its effect was reversed and lipid contents increased (52.71 FI; P<0.05). None of the stimulators of cGMP synthesis affected the expression of PLIN2 in cumulus cells. In conclusion, stimulation of sGC for cGMP synthesis promotes lipolytic activities in bovine oocytes matured *in vitro* and such effect is mediated by PKG. However, such effect may vary depending on the stimulus received and/or which synthesis enzyme was activated, as stimulation of mGC had no effects.

## Background

Throughout the years, the *in vitro* production of embryos (IVP) has been increasing its importance as an assisted reproduction technology and is employed worldwide in animal production to accelerate the production of genetically superior animals (Perry, 2015). However, bovine oocytes and blastocysts produced *in vitro* are inferior in quality compared to those produced *in vivo* as they present more swollen blastomeres, slower growth rate (Fair et al., 2001), morphological, biochemical and metabolic alterations (Lonergan et al., 2006), and darker cytoplasm as a consequence of higher lipid accumulation (Pollard and Leibo, 1994). This last characteristic has been associated with lower quality of embryos produced *in vitro* and to their higher sensitivity to cryopreservation (Rizos at al., 2002). The processes of lipogenesis (biosynthesis, incorporation or accumulation of triacylglycerols; TAG) and lipolysis (sequential breakdown of TAG into fatty acids and glycerol) occur in all cells (Haemmerle et al., 2006) and the balance between them is important for cell function (Haemmerle et al., 2006; Miyoshi et al, 2008). Therefore, studies in this area in oocytes and embryos are interesting for a better understanding of these processes in such cells, as they may lead to improved culture systems for better quality and cryotolerance of IVP embryos, which are important to ensure the trade and preservation of genetics in various species.

In human adipocytes, cyclic guanosine 3'5'-monophosphate (cGMP) signaling through the activation of the cGMP-dependent protein kinase (PKG), phosphorylates important proteins, such as hormone sensitive lipase (HSL) and perilipins (PLIN), which are involved in lipolytic activities (Langin, 2006). Therefore, cellular lipolysis can be mediated via GMPc/PKG (Sengenès et al., 2003), besides the classical cAMP/PKA pathway (Lafontan et al, 2008; Moro et al, 2007; Sengenès et al., 2000).

Cyclic GMP is recognized as an important secondary messenger of extracellular signals from nitric oxide (NO) or natriuretic peptide (NPs), which stimulate guanylate cyclase (GC) enzymes to synthesize the nucleotide (Kuhn, 2003; Kuhn et al., 2004). NO, generated through the nitric oxide synthase (NOS) pathway (Rettori and McCann, 1998), which has been identified in several tissues of the reproductive system (Sessa et al., 1994), is responsible for the activation of soluble GC (sGC), which in turn leads to cGMP synthesis (Engeli et al., 2012). NPs activate enzymes that synthesize cGMP, which are known as membrane GC (mGC) (Kuhn, 2003; Kuhn et al., 2004). These enzymes, also known as NP receptors (NPRs), have been identified in bovine cumulus-oocyte complexes (COCs) (Cesaro et al., 2015). The NP pathway is constituted by endogenous peptides involved in several actions (Misono et al., 2011), including lipolytic functions (Engeli et al., 2012) which are identified as the NP precursor type A (NPPA), type B (NPPB) and type C (NPPC) (Lafontan et al, 2008; Engeli et al., 2012; Moro and Lafontan, 2013). NPPC has been shown to participate in the control of oocyte meiotic maturation (Cesaro et al., 2015; Franciosi et al., 2014).

Most of the enzymes involved in the synthesis and also in the degradation of cGMP are expressed in bovine oocytes and cumulus cells (Cesaro et al., 2015; Schwarz et al., 2014). Recently, a study using sildenafil, a selective inhibitor of phosphodiesterase 5 (PDE5), the enzyme responsible for the hydrolysis of cGMP (Francis et al., 2011), to increase cGMP levels, reported the reduction in lipid content in bovine oocytes matured in the presence of fetal calf serum (FCS) (Schwarz et al., 2017). This study indicates the involvement of cGMP, possibly through PKG activation, in the lipolysis process in bovine COCs, as reported in adipocytes (Langin, 2006).

Aiming to improve the knowledge on the involvement of this pathway in lipolytic activities in bovine CCOs, the aim of the present study was to investigate the effects of different stimulators of cGMP synthesis on oocyte lipid contents. Additionally, the relative expression of transcripts for perilipin 2 (PLIN2), a protein participating in the control of lipolysis (Lolicato et al., 2014), was evaluated in cumulus cells, as they also influence oocyte lipid metabolism (Auclair et al., 2013) and are also influenced by culture conditions that increase lipids in oocytes (Schwarz et al., 2018).

## Methods

### Media and chemicals

Chemicals were purchased from Sigma Chemical (St Louis, MO, USA), unless otherwise stated.

### Ethics approval

This work was approved by the Ethics Committee on the Use of Animals (Protocol 3171160518 - CEUA), Faculdade de Zootecnia e Engenharia de Alimentos, USP, Pirassununga, SP, Brazil.

### Cumulus-oocyte complexes collection

Bovine ovaries were collected at a commercial abattoir immediately after slaughter and transported in sterile saline solution with antibiotics (100 IU/mL penicillin and 100 mg/mL streptomycin) at 30°C. In the laboratory, 2 to 8 mm follicles were aspirated with an 18 “G” needle attached to a disposable 10 mL syringe. The aspirated follicular fluid was placed in 50 mL conical tubes and maintained for 5 min for sedimentation. The upper portion of the liquid was removed and the remaining portion was added with 3 to 5 mL washing medium [TCM199 with 25 mM Hepes, 10 μg/mL gentamycin and 1% fetal calf serum (FCS, Gibco-BRL, Grand Island, NY,USA)]. The material was then transferred to a Petri dish (60 x 15 mm) under a stereomicroscope for selection of grade I and II cumulus-oocyte complexes (COCs) (De Loos et al., 1991).

### *In vitro* maturation

For *in vitro* maturation (IVM), the selected COCs were cultured in maturation medium [TCM199 with 20 mM bicarbonate containing 0.2 mM sodium pyruvate, 0.5 μg/mL follicle stimulating hormone (FSH) and 10 μg/mL gentamicin] with 0.1% polyvinyl alcohol (PVA) or 10% FCS, according to the experiment. Groups of 25 COCs were randomly distributed into droplets of each treatment (100μL maturation medium under mineral oil) and incubated at 38.5°C and 5% CO_2_ in air and maximum humidity for 24 h.

### Separation of cumulus cells and oocytes

Groups of 25 COCs were denuded by pipetting in Ca^2+^Mg^2+^-free phosphate buffered saline + 0.1% polyvinyl alcohol (PBS + PVA). The partially denuded oocytes (DOs) were removed from the solution and vortexed for 4 min in PBS+PVA to remove any remaining cumulus cells (CC). After washing in PBS+PVA, DO pools were evaluated for nuclear maturation and lipid content. The solution containing the CC was centrifuged at 900 X g for 10 min. The supernatant was removed, and the cell pellet treated with 1.0 U/μL RNase OUT and stored at -80^o^ C for later evaluation by quantitative Real-Time PCR.

### Intracellular lipid staining

In the study, three different lipid stains, Nile Red (Huang et al., 2009), BODIPY (Listenberger et al., 2016) and Sudan Black B (Sudano et al., 2012), were compared in order to verify which one would be more efficient for measuring lipid content in bovine oocytes.

### Nile Red

DOs (5 replicates/group) were washed in PBS+PVA, fixed in 4% paraformaldehyde (PFD) and simultaneously permeabilized with 0.5% Triton X-100 solution in PBS+PVA for 15 min at room temperature and then washed again 3x in PBS+PVA. Oocytes were stained with 1 μg/mL Nile Red (N3013 – Molecular Probes, Life Technologies, Eugene, USA) in PBS+PVA for 30 min, protected from light and at room temperature. After this period, the oocytes were washed 3x in PBS+PVA and transferred to a glass slide (12 oocytes/slide) containing 5μL Pro Long Gold (Invitrogen), which was carefully covered with a coverslip. The fluorescence of Nile Red was evaluated under an epifluorescence microscope [Nikon Eclipse - TS100/filter G2A; excitation 515 to 560 nm and emission greater than 590 nm (Greenspan et al., 1985) with fast resolution (Romek et al., 2011)]. All the photos obtained had their fluorescence intensities measured by the ImageJ software (Wayne Rasband, National Institutes of Health, Bethesda, MD). The photos were converted to gray scale (8 BIT) and the area of the oocyte to be analyzed was delimited. The lipid content in oocytes is presented as mean fluorescence intensity (FI) (Fu et al., 2011).

### Bodipy 493/503

DOs (5 replicates/group) were washed in PBS+PVA and fixed in 4% PFD for 30 min. Then, they were permeabilized with 0.1% saponin in PBS+PVA for 60 min at room temperature and then washed again 3x in PBS+PVA. Oocytes were stained with 20 μg/mL Bodipy 493/503 (D3922 – Molecular Probes, Life Technologies, Eugene, USA) in PBS+PVA for 60 min, protected from light and at room temperature. After this period, oocytes were prepared as previously, but place in 5 μL Fluoromount G instead of Pro Long Gold. The fluorescence of Bodipy was evaluated under the same microscope, but using eGTP filter, 500 to 530nm excitation and emission greater than 503 nm. Lipid content was measured as for Nile Red staining (Fu et al., 2011).

### Sudan Black B

DOs (5 replicates/group) were washed in PBS+PVA and fixed in 10% formalin solution for 2 hours. Next, they were washed 3x in distilled water with 0.05% PVA. Then, DOs were transferred a 50% ethanol with 0.05% PVA solution for 2 minutes, followed by staining in 1% Sudan Black B in 70% ethanol solution with 0.5% PVA for 4 minutes protected from light. Next, the oocytes were washed 3 times in 50% ethanol and 2 times in distilled water with PVA. The stained oocytes were transferred to a glass slide (12 oocytes/slide) and carefully covered with a coverslip and then submitted to optical microscope evaluation. All the photos obtained had their gray and black pigments measured by the ImageJ software. The photos were converted to gray scale (8 BIT) and the area of the oocyte to be analyzed was delimited and gray intensity per area was calculated (arbitrary unit/ µm^2^) (Sudano et al., 2012).

### Determination of nuclear maturation

Dos (5 replicates/group) were stained with 10 μg/mL Hoechst 33342 (Invitrogen) for 15 min protected from light and at room temperature. According to the experiment, oocytes were washed 3x in PBS+PVA and stained with Hoechst 33342 (H1399 – Invitrogen, Thermo Fisher Scientific) and Nile Red, simultaneously. The fluorescence for Hoechst 33342 was evaluated under the same epifluorescence microscope using AT-DAPI filter, excitation 475 to 490nm and emission 445 to 450nm. The percentage of oocytes that reached metaphase II was determined as the proportion of cultured oocytes presenting extrusion of the second polar body.

### Quantitative real time polymerase chain reaction (qPCR)

All CC samples (5 replicates/group = 5 sample/group) collected after IVM were submitted to total RNA extraction according to the Trizol® protocol (InvitrogenTM). After extraction, total RNA samples were quantified by spectrophotometry using a NanoDrop® 2000 (Thermo Scientific) to verify integrity assessing the 260/280 ratio (between 1.8 and 2.0). For DNA digestion and reverse transcription procedures CC samples had their volumes adjusted to contain 1.000 ng RNA per sample. All samples were submitted to DNA digestion procedure using the enzyme DNAse I - Amplification Grade® (Invitrogen). For reverse transcription (RT) and synthesis of complementary DNA (cDNA), the “High Capacity cDNA Reverse Transcription” kit was used (Applied Biosystems Carlsbad, CA-USA). Relative quantification of transcribed genes was performed by real time 166 qPCR, using the SybrGreen® detection system with Power SybrGreen® PCR Master Mix reagent (Applied Biosystems TM), in the Applied Biosystems Step One equipment. qPCR reactions were run in 12 μL containing 0.25 mM of each primer, 1X SYBR Green PCR Master Mix (Applied Biosystems), 2.5 μL H_2_O and 2.0 μL template (two-fold diluted cDNA; 50ng). Cycling conditions for amplification were: 95^o^C for 10 min followed by 45 cycles at 95^o^C for 15 sec, 57^o^C for 20 sec and 60^o^ C for 40 sec. Each sample was analyzed in duplicate for each of the genes. As a negative control for the reaction, ultrapure DNAse and RNAse free water was used in place of cDNA. Values of expression of target gene PLIN2 were normalized by the geometric mean of mRNA transcripts of two housekeeping genes, GAPDH and ACTB (Lonergan et al., 2003). Differences in frequencies of transcripts were calculated by the 2^-ΔΔCt^ comparative method (Livak and Schmittgen, 2001). A “threshold” line was fixed at the mean point of the exponential amplification to each gene. Primers were designed (Table 1), based on the bovine sequences available on the genome browser of the University of California Santa Cruz (UCSC), using the Primer3 software (Primer3web, version 4.0.0).

**Table 1:** Sequence of initiator oligonucleotides (primers) used for relative quantification of transcripts for the selected genes.

**Gene**	**Primer Sequence (5’ – 3’)**	**GenBank**	**Size (bp)**
*perilipin 2*	5’ ATGAATCCCACTGTGCTGAG 3’	NM_174417.2	135
*(Plin 2)*	5’ CCCGATCTTGAATGTTCTGTG 3’		
*actin beta*	5’ GGCACCCAGCACAATGAAGA 3’	NM_173979.3	67
*(Actb)*	5’ GCCAATCCACACGGAGTACTT 3’		
*glyceraldehyde-3-phosphate dehydrogenase*	5’ CCACTCCCAACGTGTCTGTT 3’	NM_001034034.2	84
*(Gapdh)*	5’ GCTTCACCACCTTCTTGATCTCATC 3’		

### Statistical analysis

Statistical analyses were performed using the SAS System (V 5.1; SAS Institute, Inc., Cary, NC), by one-way ANOVA followed by Tukey post hoc test. Data were tested for normal distribution and homogeneity of variance and were transformed to arcsine (maturation rate) or log10 (lipid content) when these criteria were not met. For gene expression the values of 2^-ΔΔct^ were considered (Livak and Schmittgen, 2001). Data for 5 replicates/experiment are presented as mean ± SEM. Differences with probabilities of P <0.05 were considered significant.

## Results

### Comparative assessment of Nile Red, Bodipy 493/503 and Sudan Black B staining for lipid content in oocytes

**Figure 1:**
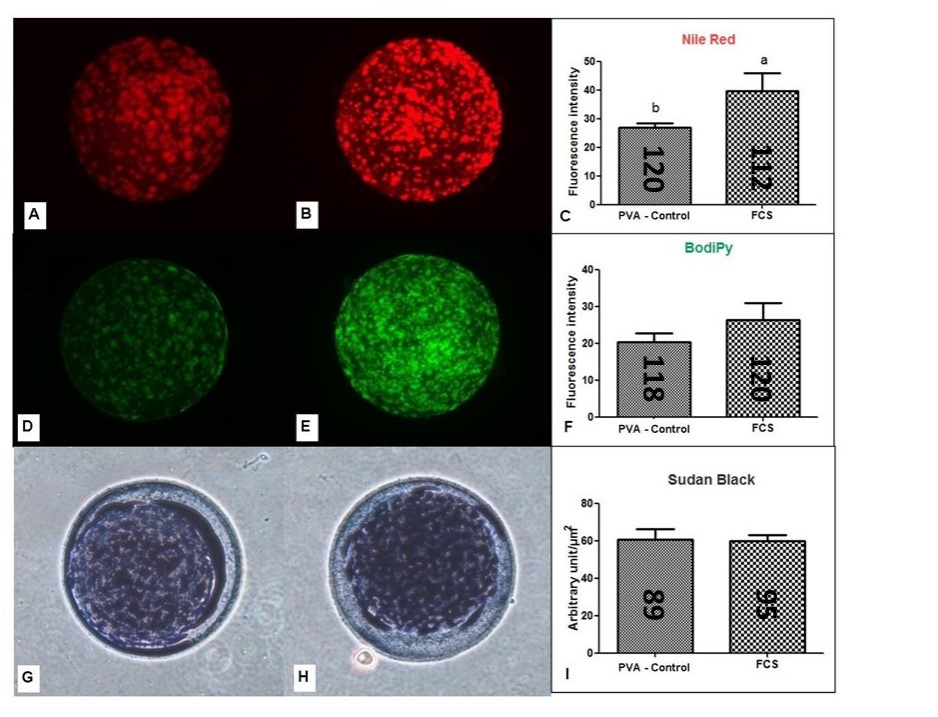
Total lipid content in bovine oocytes determined by different staining methods. **A and B:** Representative images of oocytes stained with Nile Red, **D and E**: with Bodipy 493/503, **G and H**: with Sudan Black B, being respectively controls (PVA, **A, D and G**) and FCS (**B, E and H**). **C, F and I**: Fluorescence or pigment intensity measured with appropriate filters. The numbers inside the columns indicate the number of oocytes evaluated per treatment. Data are expressed as the mean ± SEM of five replicates. Values with different superscript letters differ significantly (P <0.05). FI - fluorescence intensity, AU - arbitrary unit/µm^2^.

Three commonly used stains for lipid measurement, Nile Red, Bodipy 493/503 and Sudan Black B, were analyzed in oocytes submitted to IVM, to compare the results and select the most appropriate staining technique. Oocytes from COCs matured in the presence of FCS had a higher lipid content (39.73 FI) compared to the control group (PVA, 26.96 FI; P<0.05) when evaluated by the Nile Red (Figure 1). This difference was not detected in the groups evaluated with Bodipy 493/503 (26.25 FI for FCS and 20.35 FI for control, P>0.05) or Sudan Black B (59.67 AU in FCS and 60.55 AU in control; P>0.05). Considering the criteria of data repeatability, runtime and protocol ease, lipid staining by Nile Red seemed to be more efficient, and was therefore used for the next experiments.

### Effect of stimulation of different cGMP synthesis enzymes on nuclear maturation in IVM oocytes

Different agents to stimulate cGMP synthesis were tested for oocyte nuclear maturation after the IVM period, to assure treatments would not negatively interfere on oocyte nuclear maturation. Only the presence of NPPC reduced the number of COCs that completed metaphase II, with a maturation rate of 68.3% MII in relation to the control (77.7% MII, P>0.05). GC stimulation by Protoporphyrin IX (for sGC) or NPPA (for mGC), did not influence nuclear maturation (75.9 and 79. 5% MII, respectively), which was similar to the control group (P>0.05; Table 2).

**Table 2:** Nuclear maturation rates of oocytes submitted to *in vitro* maturation for 24 hours.

**Treatments**	**Oocytes** **(n)**	**Maturation Rate** **(%MII ± SEM)**
FCS (control)	112	77.7 ± 5.3^a^
Protoporfyrin IX	106	75.9 ± 4.9^a^
NPPA	108	79.5 ± 4.0^a^
NPPC	134	68.3 ± 0.1^b^

Different letters within the column indicate significant difference (P<0.05). Results from 5 replicates. Means presented with mean standard error.

### Effect of stimulation of different cGMP synthesis enzymes on lipid content in IVM oocytes

**Figure 2:**
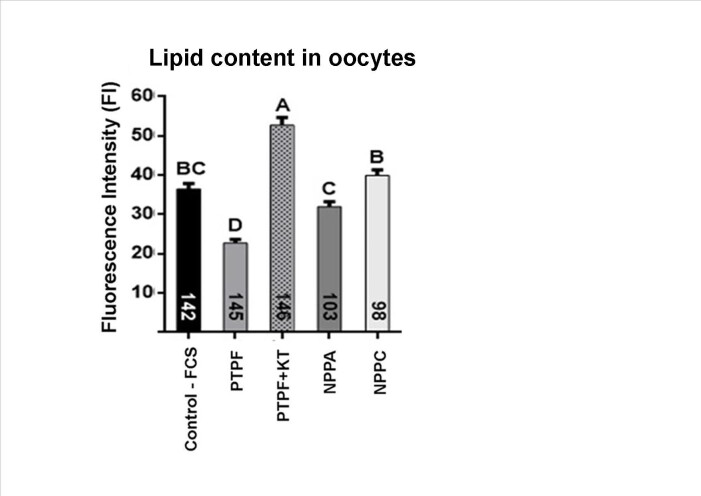
Lipid content in bovine oocytes derived from COCs matured *in vitro* for 24h with different stimulators of cGMP synthesis enzymes. Control: FCS only. Treatments: Protoporphyrin IX (PTPF), PTPF associated with PKG inhibitor KT5823 (KT), NPPA and NPPC. The numbers inside the columns indicate the number of oocytes evaluated per treatment. Columns with different letters indicate significant difference (P<0.05). Results from 5 replicates.

The stimulation of sGC by Protoporphyrin IX generated oocytes with the lowest lipid content (22.7 FI), in relation to the other treatments (NPPA 31.99 FI and NPPC 39.91 FI) and to the control (36.45 FI, P<0.05; Figure 2). However, when KT5823 (PKG inhibitor) was associated with Protoporphyrin IX, this effect was reversed (52.71FI, P<0.05), suggesting that the observed lipid reduction could be under cGMP influence, as its levels were supposedly elevated by stimulation of sGC, leading to increased PKG activity. As mentioned, the other cGMP modulators (stimulating mGC), at the concentrations studied, had no effect on lipid content. Thus, cGMP appears to interfere differently with lipolytic activities in the oocyte depending on the stimulus received for its synthesis.

### Assessment of PLIN2 transcripts in cumulus cells

**Figure 3:**
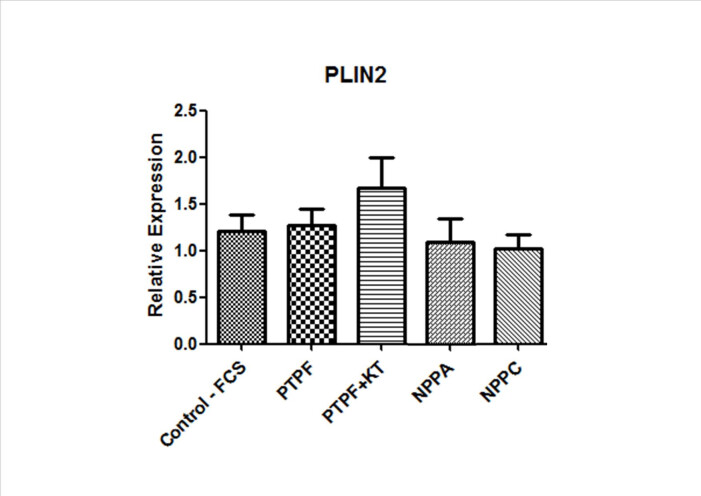
Relative abundance of the PLIN2 transcript in cumulus cells, originating from 25 COCs/group matured *in vitro* for 24h in the presence of different stimulators of cGMP synthesis enzymes. Control: FCS. Treatments: Protoporphyrin IX (PTPF), PTPF associated with KT5823 (KT), NPPA and NPPC. Results from 5 samples/group (5 replicates).

As already mentioned, cumulus cells influence oocyte lipid metabolism (Auclair et al., 2013) and are influenced by culture conditions that increase lipids in oocytes (Schwarz et al., 2018). Also, as PLIN2 has been associated with accumulation of lipids its transcript relative abundance in cumulus cells was assessed under the influence of different cGMP synthesis stimulators during IVM (Figure 3). None of the GC stimulators used interfered with the expression of PLIN2 and were all similar to the control group (P>0.05).

## Discussion

The present study had as main objective to investigate the action of the stimulation of enzymes that synthesize cGMP on lipid contents in bovine COCs matured *in vitro*. The accumulation of cytoplasmic lipid has been pointed out as one of the characteristics related to the reduced quality of the embryo produced *in vitro* in relation to those produced *in vivo* (Rizos et al., 2002; Ambruosi et al., 2009).

Initially a comparison of different methodologies for the detection and measurement of cytoplasmic lipids was made. In this work, the three lipid staining techniques most commonly used were compared in denuded oocytes after the *in vitro* maturation. Lipid detection by Nile Red appeared to be more efficient because it captured the increase in lipid content that occurs when COCs are matured in culture medium enriched with FCS (Del Collado et al., 2014) in relation to the control constituted by PVA, which is an alcohol that only increases the surface tension without exerting any function on lipolytic activities (Asada et al., 2002). This technique was previously validated in bovine oocytes as being efficient for detection of variations of lipid content in different culture conditions (Genicot et al., 2005; Barceló-Fimbres and Seidel, 2011). In view of the results, Nile Red staining was selected for the other experiments, since in under the experimental conditions used it was the most practical and sensitive for assessing lipid content in oocytes.

Considering that the cGMP level interferes with the resumption of meiosis of mouse (Törnell et al., 1990) and bovine oocytes (Törnell et al., 1990; Bilodeau-goeseels, 2007; Schwarz et al., 2014), the interference of the drugs used in the present study, which affect cGMP levels, was evaluated regarding the progression of meiosis to metaphase II. The results showed that only the group treated with NPPC presented a reduced maturation rate in relation to the others. There are few studies with NPs in cattle, but previous studies have shown a reduction in the resumption of meiosis with NPPC (Franciosi et al., 2014; Cesaro et al., 2015) and also with NPPA (Cesaro et al., 2015), however, neither study evaluated progression to MII.

In a previous study in COCs treated with Protoporphyrin IX, a delay in meiosis resumption was also observed, but when the progression to MII was evaluated after 24 h of IVM, there was no reduction (Schwarz et al., 2014). Thus, our results corroborate previous observations that cGMP generated by Protoporphyrin IX, although affecting meiosis resumption, has no negative effect on the progression of nuclear maturation. It is possible that a similar phenomenon occurs with NPPA, because in this study it did not affect maturation up to MII, although it reduced meiosis resumption in a previous study (Cesaro et al., 2015). In the study by Schwarz et al (2014), a cGMP analogue and a NO donor had the same effect (reduction in resumption and normal progression), suggesting that cGMP affects GVBD kinetics, but not further meiosis progression. The case of NPPC, however, seems to indicate that it is actually more involved in the control of meiosis as observed in mouse (Zhang et al., 2010). Besides, Franciosi et al (2014) and Cesaro et al (2015) showed reduction in meiosis resumption and in the present study there was also a reduction in final maturation. More studies are needed to address the role of NPPC and cGMP generated by this peptide on the control of nuclear maturation.

As in adipocytes the elevation of cGMP levels favors lipolysis (Sengenès et al., 2003), we evaluated whether stimulating the nucleotide synthesis would also affect lipid metabolism in bovine COCs. According to our results, only Protoporphyrin IX reduced the lipid contents in oocytes after IVM, suggesting that the stimulation of cGMP synthesis by sGC leads to the activation of a lipolytic process. The action of cGMP occurs more frequently by the activation of PKG1 and PKG2 (Lohmann et al., 1997), which can phosphorylate important proteins involved in lipid metabolism, such as HSL and some perilipins (Sengenès et al., 2003). When PKG was inhibited by associating KT5823 with Protoporphyrin IX, the reduction of lipid content was reversed pointing to a mechanism of action of cGMP through the PKG activation. As bovine oocytes express PKG1 and PKG2 (Schwarz et al., 2014, 2017), the action of this pathway in these cells is plausible.

However, the stimulation of lipolysis by the elevation of cGMP through NPs reported in adipocytes (Lafontan et al., 2008; Engeli et al., 2012; Moro and Lafontan, 2013), was not mimicked in oocytes. In the present study, a reduction in the lipid content of oocytes matured with NPPA or NPPC was not observed. The reasons for the lack of effect are not known and this type of evaluation has not been done previously in these cell types. It is known, however, that NPPA and NPPB have higher affinity for the NPR1 receptor and NPPC for the NPR2 receptor (Potter et al., 2009). Both receptors are stimulators of mGC to increase cGMP synthesis (Huang et al., 2015) and, in cattle, their transcripts were detected in cumulus cells, but in oocytes only NPR2 was found (Cesaro et al., 2015). Thus, COCs would be able to respond to the stimulation of NPs, since receptors are present, however, oocytes do not appear to have NPR1 to respond to NPPA, so there was no effect of the treatment. However, although oocytes express NPR2 and could respond to NPPC stimulation, this seems not to be related to lipolysis, but to maturation control, as observed in this and in other studies (Cesaro et al., 2015; Franciosi et al., 2014). In addition, CC present NPR1 to respond to NPPA (Cesaro et al., 2015), but the evaluation of lipid content in the CC was not performed to confirm whether they would activate lipolysis. This possibility remains open and should be investigated in the future. There is also the possibility that the concentration of NPs was insufficient to stimulate lipolysis in oocytes. The dose used was based on studies with adipocytes, where concentrations of 10 to 1000nM were used, and 10nM were sufficient to stimulate a response (Sengenès et al., 2000). In oocytes, we used the highest concentration (1000nM), but no effect on lipid content. Cesaro et al. (2015) measured cGMP in oocytes and bovine COCs and observed that there was elevation in nucleotide levels, suggesting that this dosage would be sufficient to stimulate cGMP synthesis, but the authors did not test NPPA and NPPC alone, but only in combination with forskolin and only in the initial hours of maturation (3 and 6 h). The response of COCs and oocytes to the stimulation of NPs in relation to cGMP levels is not known and should be the object of future studies. There may be a distinct responsiveness between different cell types (oocytes x adipocytes).

A curious observation was that using different agents that stimulate cGMP synthesis by different enzymes, there was also a different response. That is, Protoporphyrin IX stimulating sGC stimulated lipolysis, while NPPA or NPPC stimulating mGC (NPR1 and NPR2, respectively) had no effect. It would be interesting to determine whether the level of cGMP synthesis elevation is distinct for the different stimuli to confirm whether their origin (sGC or mGC) has different results. It has been reported that different GC can be compartmentalized in different regions in the cells, generating different responses to the same signal. For example, in cardiomyocytes, cGMP levels are controlled in the plasma membrane via mGC synthesis and degradation by PDE2, while in the cytoplasm the synthesis is performed by sGC and degradation by PDE2 and PDE5 (Francis, et al. 2011). In oocytes, this uniqueness of action and control is still little known. A study by Sasseville et al. (Sasseville et al., 2008) on porcine oocytes suggested that PDE6 activity would be bound to cell membrane fractions and PDE5 to cytoplasm, and that both cGMP ​​degrading enzymes would contribute differentially for the reduction in nucleotide levels. Therefore, this area requires more studies.

Once there was variation in the lipid content of the oocytes caused by the different treatments and CC express NPR1 and NPR2 to respond to NPs and also sGC to respond to Protoporphyrin IX (Schwarz et al., 2014, 2017), the expression of transcritps for PLIN2 was evaluated in CC. PLIN2 is a protein associated with lipid accumulation by controlling the interaction between lipases and lipid droplets (Bickel et al., 2009). This protein has already been identified in CC (Lolicato et al., 2014) and an increase in the expression of this PLIN in CC has been reported when there is a higher lipid content in the oocyte (Schwarz et al., 2017, 2018). The interaction of cumulus cells can directly influence oocyte lipid metabolism (Auclair et al., 2013) since there is important communication during the first hours of IVM between oocyte and CC (Del Collado et al., 2017). Thus, we investigated the effect of different cGMP modulators on this transcript in cumulus cells. Although we detected effects of Protoporphyrin IX on oocyte lipid content, none of the stimulators of cGMP synthesis interfered with the expression of PLIN2. Previous reports observed an increase in PLIN2 transcript abundance caused by FCS in cumulus cells (Schwarz et al., 2017, 2018; Del Collado et al., 2016), while a reduction was observed when a PDE5 inhibitor (sildenafil) to inhibit the degradation of cGMP and maintain its intracellular levels was added to serum-containing medium (Schwarz et al., 2017, 2018). This variation in PLIN2 expression in CC paralleled observations in lipid content in oocytes. In the present study no changes in PLIN2 were observed. The reasons for this discrepancy are unclear, but may be a consequence of different treatments used; sildenafil in Schwarz et al. (2017, 2018) and Protoporphyrin IX in the present study. Other studies are therefore necessary to evaluate the relationship of such treatments with expression of PLIN2. In addition, it would also be interesting to evaluate if there would be any effect on the expression of the PLIN2 protein or other PLINs involved in the lipolysis process, besides the direct quantification of lipids in these cells.

## Conclusions

The cGMP/PKG pathway appears to affect lipolytic activities in bovine oocytes matured *in vitro*, however, the magnitude of such interference varies depending on the stimulus received. Lipolysis was positively affected when cGMP synthesis was stimulated by Protoporphyrin IX and this effect was reversed with inhibition of PKG suggesting the influence of PKG activity in the process. Considering the distinct mechanisms that control cGMP levels and responses, more studies are needed to understand its mechanisms on oocyte lipolysis.
